# Co-Expression Network Analysis of Fbxw7-Associated LncRNAs Reveals Their Functions in Radiation-Induced Thymic Lymphoma

**Published:** 2016-04-30

**Authors:** Antoine M Snijders, Jian-Hua Mao

**Affiliations:** Biological Systems and Engineering Division, Lawrence Berkeley National Laboratory, Berkeley, California, USA

**Keywords:** Long non-coding RNA, Thymic lymphoma, FBXW7, Radiation

## Abstract

*FBXW7,* an E3-ubiquitin protein ligase in SCFs (SKP1-cullin-F-box) complex, is a major human tumor suppressor gene, and understanding mechanisms by which FBXW7 contributes to tumorigenesis is critical for the treatment of human cancers with *FBXW7* deficiency. Long non-coding RNAs (lncRNAs) have emerged as key regulators of various biological processes. Here we have identified a set of lncRNAs that are associated with Fbxw7 deficiency. The correlation network and functional annotation analysis revealed that Fbxw7-associated lncRNAs regulate genes involved in cell cycle, DNA repair, metabolic process, and cell communication and adhesion. The number of coding genes that correlated with individual lncRNAs varied largely. A lncRNA on chromosome 15 (A_30_P01032978), which was upregulated in tumors from Fbxw7 deficient mice was positively correlated with 15 coding genes. High expression of this 15-gene signature was associated with poor prognosis in two independent human breast cancer studies. Our results open possible new avenues to understand mechanisms by which Fbxw7 deficiency increases tumor susceptibility via the alteration of lncRNAs.

## Introduction

Long non-coding RNAs (lncRNAs), previously thought of as transcriptional noise, are emerging as key regulators of a multitude of cellular processes by taking part in epigenetic, transcriptional, and post-transcriptional regulation of gene expression [1,2]. The lncRNAs have a weaker evolutionary constraint and lower levels of expression compared to the protein-coding transcripts, but exhibit more tissue specific expression than protein-coding genes [3,4]. Recently, a number of studies have shown that lncRNA expression can be deregulated in human cancers [5,6]. As the functions of individual lncRNAs in cancer are beginning to be elucidated, they are being categorized and referred to as either tumor suppressor or oncogenic lncRNAs, in the same way as traditional protein-coding cancer genes. However, the relevance of lncRNAs and their contributions to gene expression signatures in radiation-induced thymic lymphomas in mice has not yet been characterized.

FBXW7 is emerging as a major human tumor suppressor that lies at the nexus of several pathways that control cell growth, differentiation and tumorigenesis. Deletion and/or mutations of FBXW7 have been found in cancers from a wide spectrum of human tissues [7,8]. The overall frequency of point mutation is about 6%, while the frequency of deletion is more than 30% across human cancer types [9,10], suggesting that disruption of FBXW7 may be a major feature of many human cancers. Four “hotspot” mutations (R465C, R465H, R479Q and R505C of FBXW7α), which alter the core arginine residues required for interaction of FBXW7 with its targets, have been found in human cancers [9,10]. In addition, studies have shown that loss of FBXW7 is associated with poor prognosis [11,12]. Moreover, deletion of the Fbxw7 gene in mice leads to embryonic lethality, but heterozygous mice develop normally [13,14]. Although they do not develop spontaneous tumors, we have shown that heterozygous mice are susceptible to ionizing radiation (IR)-induced tumorigenesis for a variety of tumor types [15]. The FBXW7 protein constitutes one of the four subunits of an E3-ubiquitin protein ligase complex called SCFs (SKP1-cullin-F-box), which functions in phosphorylation-dependent ubiquitination and is essential for the ubiquitination of many oncoproteins [7, 8], such as Aurora-A (AURKA) [15,16], and mTOR [17]. However, how mutation/loss of FBXW7 results in tumor development remains largely unknown.

Our previous studies showed that temporal pharmacological inhibition of the mTOR pathway was sufficient to suppress the tumor development contributed by Fbxw7 loss, suggesting that the Fbxw7-mTOR pathway plays a major role in this radiation-induced carcinogenesis mouse model [18]. We furthermore showed that tumors of Fbxw7 heterozygous mice showed deregulation of cholesterol metabolic and cell cycle related processes using transcriptional profiling. In this study, we investigated the contribution of specific lncRNAs in gene expression signatures of thymic lymphomas from Fbxw7/p53 double heterozygous (Fbxw7+/− p53+/− ) mice compared to thymic lymphomas from p53 single heterozygous (p53+/− ) mice. Our results indicate lncRNAs may exert a partial or key role in radiation-induced tumors through the regulation of mRNA expression.

## Results and Discussions

### Identification of lncRNAs associated with Fbxw7 deficiency in radiation-induced thymic lymphomas

**Figure 1:**
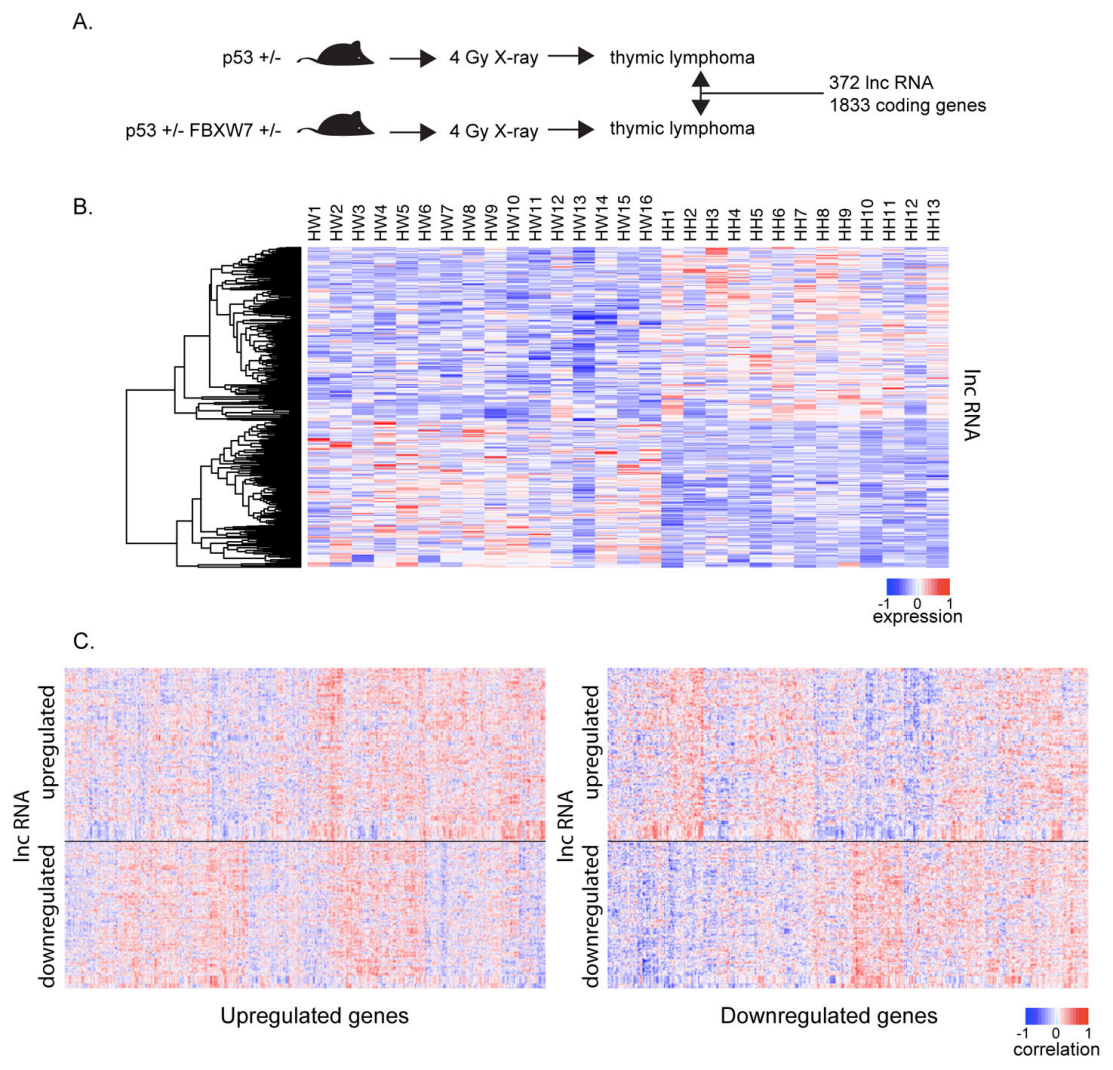
LncRNAs associated with Fbxw7 deficiency in radiation-induced thymic lymphomas. A. Schematic diagram of experimental approach. At 5 weeks of age, p53+/− and p53+/− Fbxw7+/− mice were exposed whole-body to a single dose of 4 Gy X-ray irradiation and were monitored for tumor development. Transcription profiling of radiation-induced thymic lymphomas from Fbxw7+/− p53+/− mice (n=13) or from p53 single heterozygous (p53+/− ) mice (n=16) revealed differential expression of 1833 coding genes and 372 lncRNAs (fold-change 1.3; p<0.05). B. Unsupervised hierarchical clustering of lncRNAs differentially expressed in thymic lymphomas of Fbxw7+/− p53+/− mice (HH) and p53 single heterozygous (HW) mice. C. Correlation matrix heatmap (Spearman rank correlation) of differentially expressed lncRNAs and upregulated (left) and downregulated (right) coding genes.

FBXW7 is one of the most important tumor suppressor genes in human cancer. However, the mechanisms by which FBXW7 contribute to tumor development still remain largely unclear. To gain insight into the molecular mechanisms, we identified the lncRNAs that are associated with FBXW7 deficiency, since lncRNAs have an emerging role in cancer development and progression. Therefore, we transcriptionally profiled radiation-induced thymic lymphomas from Fbxw7+/− p53+/− mice (n=13) or from p53+/− mice (n=16) using microarrays that contained both mRNAs and lncRNAs. In comparison to transcriptome of thymic lymphomas from p53+/− mice, we found 2205 differentially expressed transcripts in thymic lymphomas from Fbxw7+/− p53+/− mice (fold-change 1.3; p<0.05, Figure 1A and Table S1, S2). Seventeen percent of deregulated transcripts (372/2205) are known lncRNAs, which are defined as an Fbxw7-associated lncRNA signature. It was found that 170 of 372 lncRNAs were downregulated, whereas 202 lncRNAs were upregulated in thymic lymphomas from Fbxw7+/− p53+/− mice (Figure 1A and 1B), indicating that Fbxw7-associated lncRNAs execute different functions in tumor development, some may play a tumor suppressive role, while others have an oncogenic function.

### Strong correlation between the expression of long non-coding and coding RNAs

In order to infer the function and role of Fbxw7-associated lncRNAs in tumor development, we carried out co-expression analysis between lncRNAs and mRNAs. Correlation analysis of differentially expressed transcripts in p53+/− and Fbxw7+/− p53+/− tumors showed strong correlations between transcripts (Figure S1). To specifically address whether specific lncRNAs and coding genes were correlated in expression, we globally investigated correlations between lncRNAs and coding RNAs. This analysis revealed specific clusters of lncRNAs to be positively or negatively correlated with coding RNAs in p53+/− and Fbxw7+/− p53+/− tumors (Figure 1C and 1D; Table S3, S4). These results suggest that lncRNAs regulate coding genes via either transcriptional activation or suppression.

### FBXW7-associated lncRNAs regulate genes involved in cell cycle and DNA repair

**Figure 2:**
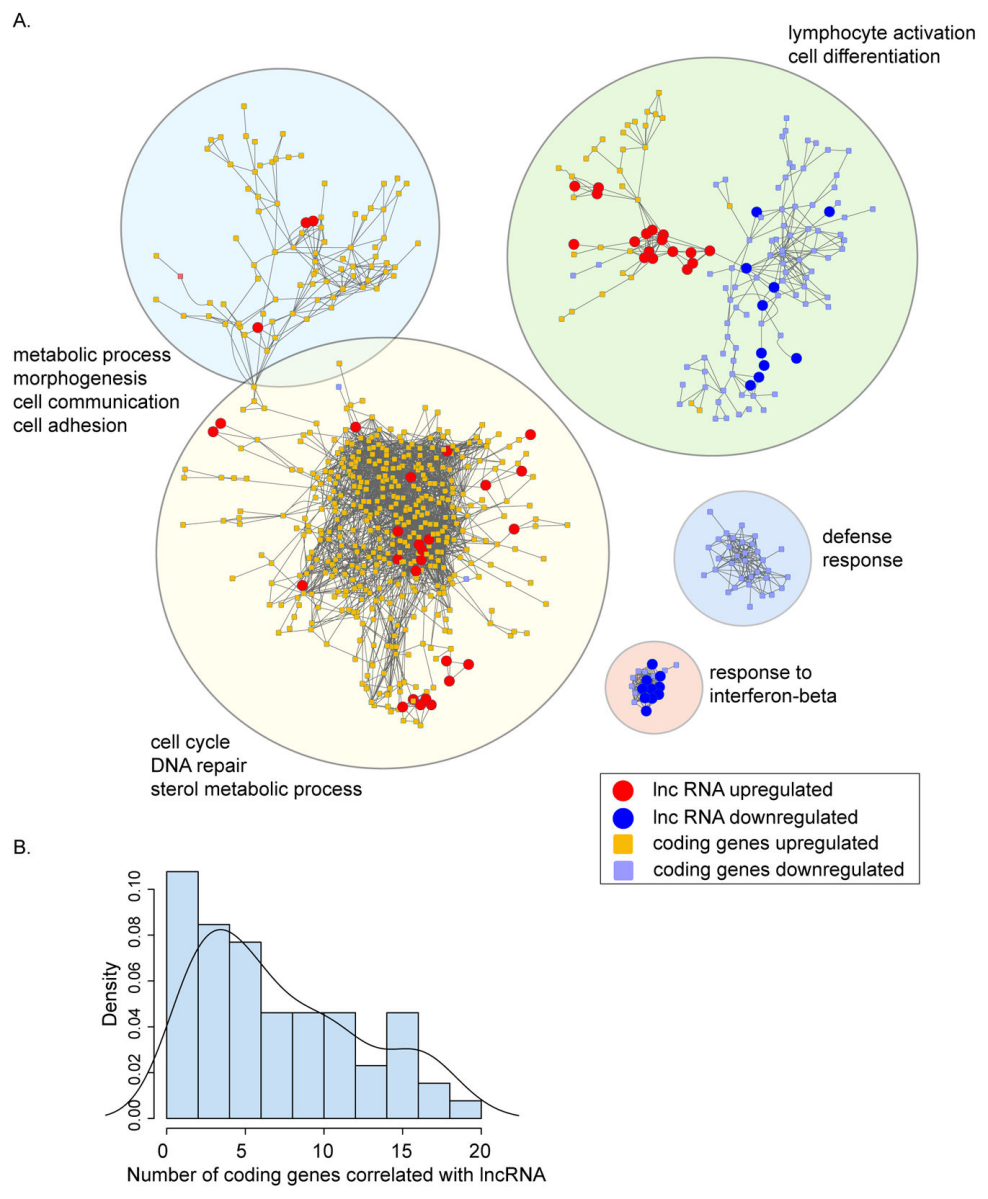
Expression correlation network of long non-coding and coding RNAs. A. Transcript correlation network of coding genes (squares: upregulated in orange; downregulated in purple) and lncRNAs (circles: upregulated in red; downregulated in blue) based on a correlation coefficient =0.8. The correlation network only includes genes and lncRNAs that were differentially expressed between Fbxw7+/− p53+/− and p53+/− tumors. Biological processes significantly enriched for each subnetwork are listed. B. Histogram and density estimate of number of coding genes correlated with lncRNAs.

To further investigate whether we could infer biologically relevant functions from the observed correlations in expression between coding RNAs and lncRNAs we generated a correlation network (correlation coefficient =0.8) based on expression levels in all tumors for 2205 differentially expressed transcripts. Five major subnetworks were observed, four of which contained lncRNAs (Figure 2A). Gene ontology enrichment analysis of coding genes within each of these subnetworks was performed to investigate if genes correlated in expression where functionally related. We observed significant enrichment for response to interferon-beta (adjusted p=8.45e–08), defense response (adjusted p=0.0061), metabolic processes, cell communication, adhesion and morphogenesis (adjusted p<0.0004), lymphocyte activation and cell differentiation (adjusted p<0.0004) and cell cycle, DNA repair and sterol metabolism (adjusted p< 2.06e-06) (Figure 2A). It is surprising that the subnetwork enriched for defense responses, containing defensin alpha 1 and three related sequences, did not contain any lncRNAs, however it remained possible that the stringent correlation coefficient prevented us from detecting any correlation. Indeed, when we lowered the stringency (correlation coefficient =0.7), we did find a number of lncRNAs that correlated with coding genes in this subnetwork (data not shown).

**Figure 3:**
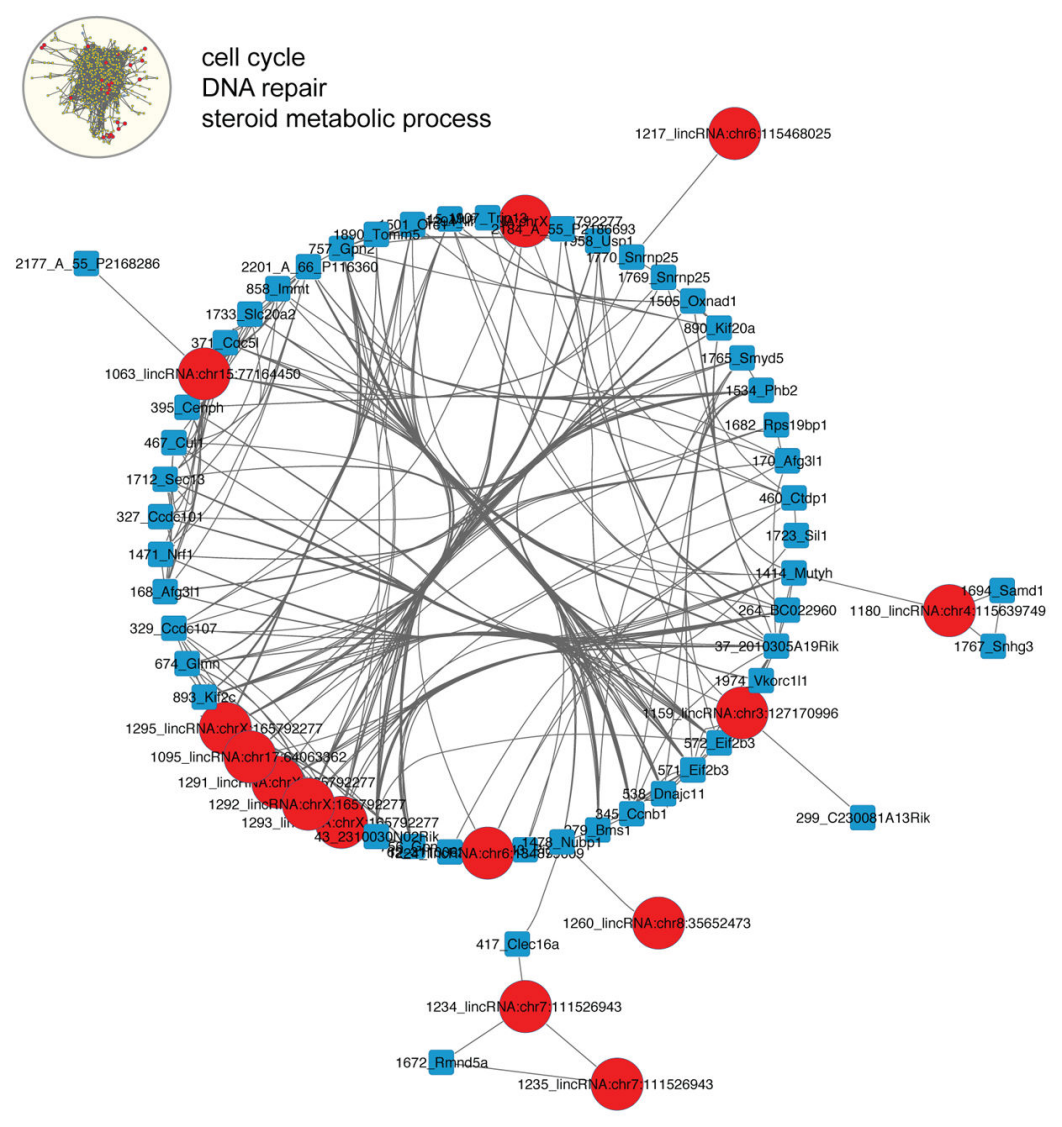
Expression correlation subnetwork significantly enriched for cell cycle, DNA repair and steroid metabolic processes. Transcript correlation subnetwork of coding genes (squares) and lncRNAs (circles) based on a correlation coefficient ≥ 0.8.

Not surprisingly, we found that the number of coding genes correlated with lncRNAs covered a wide range (Figure 2B). While the majority of lncRNAs have significant correlation with 1 or 2 coding genes, some showed significant correlation with many coding genes (Figure 2B). We then asked which genes were correlated with specific lncRNAs by selecting all lncRNAs and neighboring coding genes that correlated at the expression level. The cell cycle gene enriched subnetwork contained 16 lncRNAs, which mapped to 10 distinct genomic loci (Figure 3, Table S5).The majority of genes in this subnetwork were upregulated in tumors derived from Fbxw7+/− p53+/− mice compared to p53+/− mice indicating that the reported decreased latency observed in radiation induced tumors that arise in a Fbxw7+/− p53+/− deficient mice is at least partially mediated through increased cell proliferation mechanisms by altering lncRNA expression.

### FBXW7-associated lncRNAs regulate genes involved in lymphocyte activation and cell differentiation

**Figure 4:**
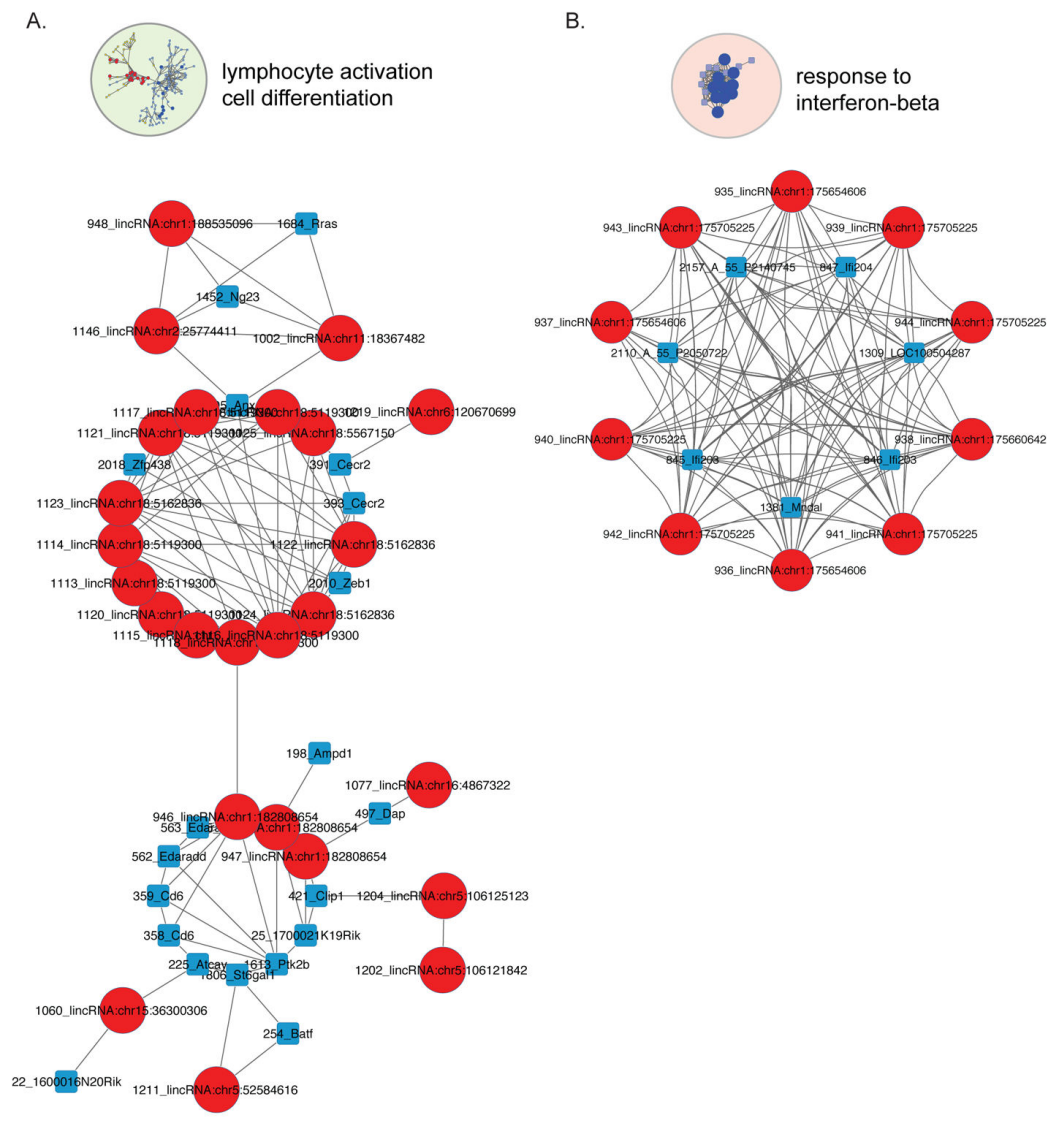
Expression correlation subnetwork significantly enriched for immune-related processes. A. Transcript correlation subnetwork of coding genes (squares) and lncRNAs (circles) significantly enriched for lymphocyte activation and cell differentiaion based on a correlation coefficient ≥0.8. B. Transcript correlation subnetwork of coding genes (squares) and lncRNAs (circles) significantly enriched for the interferon-beta response based on a correlation coefficient ≥0.8.

Two lncRNAs on chromosome 18 approximately 40 kb apart were found to be correlated with Anxa2 and Cecr2 (lncRNA position: 5119300) and Cecr2, Zeb1 and Zfp438 (lncRNA position: 5162836) (Figure 4A; Table S5) in the lymphocyte activation and differentiation subnetwork. Interestingly, Zeb1 and Zfp438 map to this same region on chromosome 18. Zeb1 was recently identified as a key regulator in a rare leukemic form of cutaneous T cell lymphoma [19]. Zeb1 has furthermore been implicated as an activator of the epithelial to mesenchymal transition, a critical process in cancer development [20]. The role of zfp438 in lymphoma development has not been clarified yet, however, a retroviral mutagenesis screen for genes involved in T-cell lymphoma development identified an insertion site near zfp438, suggesting that zfp438 plays a role in T-cell lymphoma development [21]. Anxa2, Cecr2, Zeb1 and Zfp438 were all found to be up-regulated in tumors from Fbxw7+/− p53+/− mice compared to p53+/− mice. On the other hand, a small cluster of genes correlated with a lncRNA on chromosome 1 (position: 182808654) was found to be down-regulated in tumors from Fbxw7+/− p53+/− mice compared to p53+/− mice and includes Cd6, Edaradd, Ptk2b, Ampd1, Dap and Clip1.

### FBXW7-associated lncRNAs regulate genes controlling the response to interferon-beta

The interferon enriched subnetwork contained 10 independent lncRNA probes corresponding to three closely spaced genomic loci on chromosome 1 at an interferon-inducible gene family locus (Figure 4B, Table S5). The genes correlated with these lncRNAs, I_203, I_204 and Mndal are interferon-inducible family members that map to this locus. In addition, LOC100504287 is a predicted interferon-inducible protein 203-like protein and probe IDs A_55_P2050722 (LOC677559) and A_55_P2140745 (LOC235882) are predicted to be similar to interferon-inducible proteins 204 and 203, respectively. In comparison to transcript levels of thymic lymphomas from p53+/− mice, this interferon signature is broadly down-regulated in thymic lymphomas from Fbxw7+/− p53+/− mice. Deregulation of interferon signaling pathways in cancer has been described and shown to be important in evasion of immune surveillance mechanisms. Also, an increased tumor incidence has been described in mouse models with defective interferon signaling [22,23]. Our data suggests that loss of Fbxw7 significantly deregulates interferon signaling reducing tumor latency by evading host tumor suppressor immune surveillance mechanisms.

### FBXW7-associated lncRNAs are correlated with clinical outcome in human breast cancer

**Figure 5:**
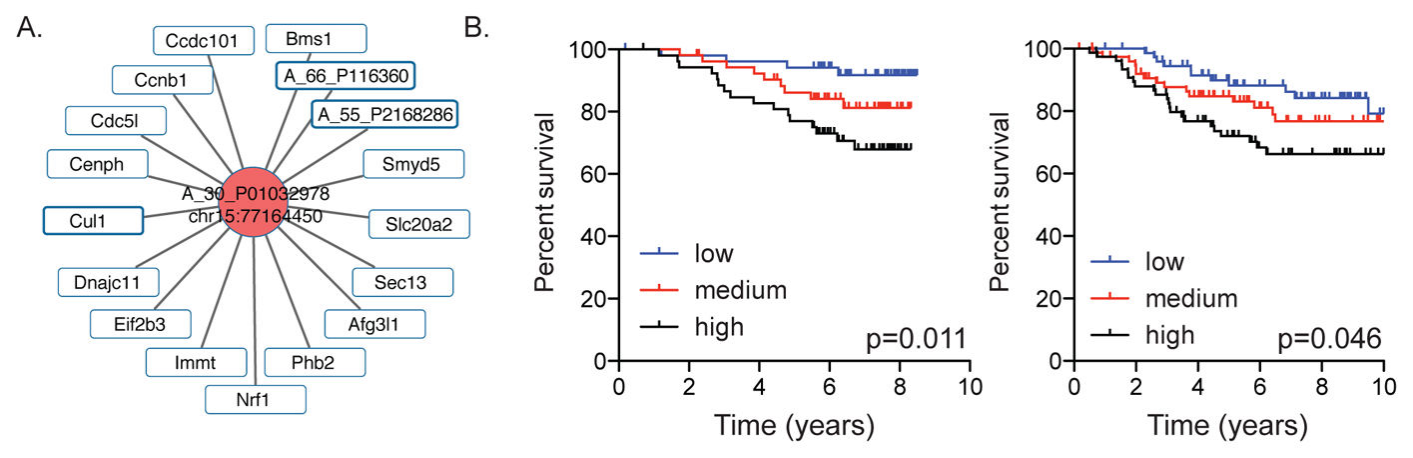
LncRNA correlated gene expression signature associated with disease free survival in human breast cancer patients. A. Genes correlated in expression with lncRNA A_30_ P01032978 on chr15. B. Increased expression of genes correlated in expression with lncRNA A_30_P01032978 is associated with decreased disease free survival in human breast cancer patients (GSE1456; p=0.011 and GSE6532; p=0.046).

To investigate if FBXW7-associated lncRNAs are relevant to human cancer, we first found human homologs of the coding genes that are significantly correlated with lncRNAs and then examine whether these human homologous genes are correlated with clinical outcome. As shown in Figure 5A, one lncRNA on mouse chromosome 15 (chr15:77164450) was significantly correlated with 17 coding genes, 15 of which have human homologs. We defined a score for each patient as the sum of the normalized expression intensities of the 15 genes. The patients were divided into three groups based on their score: the top tertile of patients with highest, middle tertile, and bottom tertile patients with lowest sum expression. We observed that patients with a higher score (top tertile) had significantly decreased survival compared to patients with a low score (bottom tertile) in a dose dependent manner (Figure 5B). This lncRNA is upregulated in tumors generated from Fbxw7 deficient mice and together with our observation that breast cancer patients with high expression of genes positively correlated with this lncRNA associated have poor prognosis, we conclude that this lncRNA likely has an oncogenic function.

In summary, our results open possible new avenues to understand mechanisms by which Fbxw7 deficiency increases tumor susceptibility via the alteration of lncRNAs.

## Materials and Methods

### Mice, irradiation and rapamycin treatment

All animal experiments were performed at Lawrence Berkeley National Laboratory and the study was carried out in strict accordance with the Guide for the Care and Use of Laboratory Animals of the National Institutes of Health. The animal use protocol was approved by the Animal Welfare and Research Committee of the Lawrence Berkeley National Laboratory. Detailed methods were described previously [18]. Briefly, p53+/− and p53+/− Fbxw7+/− mice were generated by crossing p53− /− mice with Fbxw7+/− mice. At 5 weeks of age, mice were exposed whole-body to a single dose of 4 Gy X-ray irradiation [18].

### RNA isolation and transcript profiling

Total RNA quality and quantity were determined using Agilent 2100 Bioanalyzer and NanoDrop ND-1000. Agilent SurePrint G3 Mouse GE 8x60K Microarrays were used according to the manufacturer’s protocol (arrays contained 39,430 Entrez gene RNAs and 16,251 lncRNAs). All processes were performed by Ambry Genetics (Aliso Viejo, CA). Microarray data have been deposited at NCBI GEO (accession number: GSE71975).

### Data analysis

Data normalization was performed using GeneSpring GX12.5 (Agilent Technologies). Signal intensities for each probe were normalized to the 75th percentile without baseline transformation. Genes that were differentially expressed in thymic lymphomas between vehicle and rapamycin treated Fbxw7+/− p53+/− mice and p53+/− mice were identified by the unpaired Student's t-test with a p-value cut-off of less than 0.05 and a fold change criteria of more than 1.3. Correlation analysis was performed in R using Spearman rank order correlation. Transcript correlation networks were generated using Cytoscape using the Expression Correlation Network plugin with a correlation coefficient cut-off of 0.8. Gene enrichment analysis was performed using the Gene Ontology feature of the WEB-based GEne SeT AnaLysis Toolkit [24,25].

### Human cancer datasets and survival analysis

Mouse genes that correlated in expression with lncRNA A_30_P01032978 were converted to human homologs. Gene expression microarray datasets of breast cancer for which disease-free survival was available were downloaded from the NCBI GEO website (GSE1456 and GSE6532). The sum of expression of all genes within the signature was calculated for each breast cancer patient. Kaplan-Meier plots were constructed and a long-rank test was used to determine differences among disease free survival according to the sum of the normalized expression intensities.

## Supplementary Material

TableS1

TableS2

TableS3

TableS4

TableS5
